# Effects of Trilostane on urinary Catecholamines and their metabolites in dogs with Hypercortisolism

**DOI:** 10.1186/s12917-017-1187-0

**Published:** 2017-09-04

**Authors:** Nadja Sieber-Ruckstuhl, Elena Salesov, Saskia Quante, Barbara Riond, Katharina Rentsch, Regina Hofmann-Lehmann, Claudia Reusch, Felicitas Boretti

**Affiliations:** 1 Clinic for Small Animal Internal Medicine, Vetsuisse Faculty University of Zurich, Zurich, Switzerland; 2 Clinical Laboratory, Vetsuisse Faculty University of Zurich, Zurich, Switzerland; 3 Institute of Clinical Chemistry, University Hospital Zurich, Zurich, Switzerland; 4 Dr. Quante’s current address is Peace Avenue Veterinary Clinic G/F, Hong Kong, China

**Keywords:** ACTH, Metanephrines, Pheochromocytoma, Trilostane therapy, Canine

## Abstract

**Background:**

Glucocorticoids influence the synthesis and metabolism of catecholamines (epinephrine and norepinephrine) and metanephrines (metanephrine and normetanephrine). The aim of this study was to measure urinary catecholamines and metanephrines in dogs with hypercortisolism before and during trilostane therapy.

Urine samples were collected during initial work up and during therapy with trilostane in 14 dogs with hypercortisolism and in 25 healthy dogs. Epinephrine, norepinephrine, metanephrine and normetanephrine were measured using high-pressure liquid chromatography and expressed as ratios to urinary creatinine concentration.

**Results:**

Untreated dogs with hypercortisolism had significantly higher epinephrine, norepinephrine, and normetanephrine:creatinine ratios compared to healthy dogs. During trilostane therapy, urinary catecholamines and their metabolites did not decrease significantly. However, dogs with low post-ACTH cortisol concentrations during trilostane therapy had less increased epinephrine, norepinephrine and normetanephrine:creatinine ratios compared to healthy dogs. There was no correlation of urinary catecholamines and their metabolites with baseline or post-ACTH cortisol or endogenous ACTH concentrations during trilostane therapy.

**Conclusion:**

Influences between steroid hormones and catecholamines seem to occur, as dogs with hypercortisolism have significantly higher urinary epinephrine, norepinephrine, and normetanephrine:creatinine ratios. Once-daily trilostane therapy does not lead to a significant decrease in catecholamines and their metabolites. Trilostane-treated dogs still have increased urinary epinephrine, norepinephrine and normetanephrine:creatinine ratios during trilostane therapy.

## Background

Catecholamines (epinephrine, norepinephrine) are produced by the chromaffin cells of the adrenal medulla and the postganglionic fibers of the sympathetic nervous system. They are synthesized from the amino acid tyrosine, which is converted to L-dihydroxyphenylalanine (L-DOPA), next to dopamine and finally to norepinephrine [1]. In most sympathetic postganglionic neurons, norepinephrine is the final product. In the cells of the adrenal medulla, norepinephrine is further converted into epinephrine [1]. After release into the plasma, catecholamines are quickly metabolized. Metabolism takes place mainly in the adrenal gland cells and only a small proportion in the liver and kidneys and includes the conversion of epinephrine to metanephrine and norepinephrine to normetanephrine [2]. While catecholamines are released intermittently, metanephrines (metanephrine and normetanephrine) are constantly released into the circulation and excreted in the urine [2]. This constant liberation explains why measurement of urinary or plasma metanephrines has a higher accuracy to diagnose pheochromocytoma than the measurement of catecholamines [2].

The adrenal medulla comprises about one-fourth of the adrenal mass. The rest of the adrenal mass consists of the adrenal cortex containing steroid-producing adrenocortical cells. For a long time, the two cell populations within the adrenal gland were considered as two independent endocrine systems. However, nowadays, interactions between steroid hormones and catecholamines are well known. Glucocorticoids have been shown to influence several enzymes involved in catecholamine synthesis and metabolism: first, tyrosine hydroxylase, which is the rate-limiting enzyme in catecholamine biosynthesis; second, PNMT (Phenylethanolamin-N-Methyltransferase), which converts norepinephrine to epinephrine; and third, dopamine β-hydroxylase, which hydroxylates dopamine to norepinephrine [3–7]. In vitro, glucocorticoids have been shown to increase the release of catecholamines from canine adrenal glands [8]. Therefore, it was not surprising that dogs with hypercortisolism (HC) were found to have increased concentrations of urinary epinephrine-, norepinephrine- and normetanephrine-to-creatinine ratios compared to healthy dogs [9]. However, the evolution of urinary catecholamines and metanephrines concentrations during the therapy of canine HC is unknown. Hypercortisolism is an important differential diagnosis in dogs with pheochromocytomas, as clinical signs of both diseases overlap and pheochromocytomas cannot be distinguished ultrasonographically from tumors of the adrenal cortex.

Therefore, the objective of this study was to evaluate urine concentrations of catecholamines and metanephrines in dogs with HC before and during therapy with trilostane. We hypothesized that successful therapy of HC with trilostane would lead to a significant decrease in urinary epinephrine, norepinephrine, metanephrine and normetanephrine:creatinine ratios due to the cortisol-lowering effect of trilostane.

## Methods

### Animals

Fourteen dogs with naturally occurring hypercortisolism (HC) were prospectively enrolled. Results of catecholamines and metanephrines:creatinine ratios before trilostane therapy of five dogs had been previously reported as part of a former study [10]. Nine dogs were male (4 castrated) and five dogs female (4 spayed). Breeds included Dachshund (1), Giant Schnauzer (1), Nova Scotia Duck Tolling Retriever (1), Parson Jack Russell Terrier (1), Petit Bleu de Gascogne (1), Tibetan Terrier (1), Yorkshire Terrier (1), and 7 mixed-breed dogs. Age ranged between 6 and 14 years (median 8.5; standard error of mean (SEM): 0.6) and body weight between 7 and 58 kg (median 18.8; SEM: 3.7). Criteria for inclusion were the presence of clinical signs consistent with HC (e.g. polyuria, polydipsia, polyphagia, panting, skin signs, weakness, abdominal enlargement), a low-dose dexamethasone suppression test (LDDS test) or ACTH stimulation test results compatible with HC and the owner’s agreement to treat and regularly re-evaluate the dog over at least a 6-month period [9, 11]. Pituitary-dependent hypercortisolism (PDH) was diagnosed in all dogs by means of a normal or increased concentration of endogenous ACTH, bilateral symmetrical appearance of the adrenal gland determined by ultrasonography and/or demonstration of pituitary enlargement by computed tomography.

Twenty-five client-owned dogs were enrolled as controls. These dogs had been part of previous studies [9, 10, 12]. Eleven dogs were male (6 castrated) and 14 female (12 spayed), and breeds included Australian Shepherd (1), Berger Blanc Suisse (1), Bernese Mountain Dogs (2), Border Collie (1), Golden Retriever (2), Gordon Setters (2), Labrador Retriever (2), Nova Scotia Duck Tolling Retriever (1), Rhodesian Ridgeback (1), Siberian Huskies (2), Standard Poodle (1), Tervueren (1), and eight mixed-breed dogs. Age ranged between 2 and 15 years (median 7; SEM: 0.6) and body weight between 10.4 and 59.2 kg (median 26; SEM: 2.3). The dogs were considered as healthy, based on detailed information provided by their owners and the results of a physical examination, CBC, serum biochemistry profile, and urinalysis. The inclusion of the dogs in the study was approved by the veterinary office of the canton of Zurich and was in accordance with the guidelines and directives established by the Animal Welfare Act of Switzerland (TVB 199/2004).

#### Sample collecting and processing

All urine samples (healthy and sick dogs) used for analysis of catecholamines, metanephrines and creatinine were taken in hospital, either by free catch or by cystocentesis. In dogs with HC the first sample was taken in the morning during the initial work up for HC and once during trilostane therapy (at least 6 months after start of trilostane therapy). During trilostane therapy the urine was collected 2-3 h after the dose of trilostane in the morning. Urine collection and processing was done as reported previously [9, 12, 13]. Briefly, 10 ml of urine were placed in a silicone-coated tube containing 270-280 μL of 20% hydrochloric acid (HCl). Urinary pH was measured using pH indicator strips (range of pH 1 – 6) and HCl was added to achieve a pH ≤ 2 if needed. Samples were light protected and stored at −20 °C until analysis.

#### Measurement of Catecholamines and Metanephrines

Urine samples were analysed at the Institute of Clinical Chemistry, University Hospital Zurich, Zurich, Switzerland as previously described [9, 12, 13]. Urinary epinephrine, norepinephrine, total metanephrine and total normetanephrine were quantified by High Pressure Liquid Chromatography with amperometric detection as separate compounds. The terms “catecholamines” or “metanephrines” (plural form) includes epinephrine and norepinephrine or normetanephrine and metanephrine, respectively. The results were expressed as a ratio to urinary creatinine concentrations and will be listed in the following sections as: norepinephrine:creatinine, epinephrine:creatinine, normetanephrine:creatinine and metanephrine:creatinine.

#### Measurement of cortisol and endogenous ACTH

For the ACTH stimulation test, blood samples were taken before, and 60 min after, intravenous injection of 5 μg/kg synthetic tetracosactide (Synacthen®, Novartis Pharma Schweiz AG, Bern, Switzerland). Cortisol concentrations were measured by chemiluminescence assay (DPC Immulite® 1000, Siemens Schweiz AG, Zurich, Switzerland). The intra-assay coefficients of variation were 10% and 6.3% at cortisol levels of 74.5 and 521 nmol/L, respectively. The sensitivity of the assay was 5.5 nmol/L. Endogenous ACTH before ACTH stimulation was determined by a chemiluminescence assay (DPC Immulite® 1000, Siemens Schweiz AG, Zurich, Switzerland) previously validated for dogs [14, 15]. Blood was collected into chilled EDTA-coated tubes placed on ice and centrifuged at 4 °C. Cortisol and endogenous ACTH measurements were performed in house twice a week; plasma was stored either at −20 °C (cortisol) or at −80 °C (ACTH) until assayed.

#### Experimental design

The prospective study was performed between May 2006 and October 2014 at our hospital. The initial dose of trilostane for dogs with HC was 1-2 mg/kg q 24 h (in the morning). ACTH stimulation tests were performed prior to trilostane treatment (except for 1 dog) and at regular intervals thereafter (2, 4, 8, 12 and 16 weeks). The test was performed 2-3 h after the daily dose of trilostane according to the previously described treatment protocol [16]. The treatment goal was to achieve a post-ACTH cortisol concentration of 41-138 nmol/L. The dose of trilostane was adjusted in dogs with post-ACTH serum cortisol >138 nmol/L or <41 nmol/L and clinical signs suggestive of hypercortisolism (polyuria, polydipsia, polyphagia) or hypocortisolism (reduced appetite, vomiting or nausea, soft feces), respectively.

Urine samples for determination of catecholamines and metanephrines were collected at diagnosis of HC and at least 6 months after starting trilostane therapy (ensuring that all dogs were clinically controlled for at least 2 months). At the re-evaluation during trilostane therapy, dogs had to be clinically controlled (polyuria, polydipsia and panting decreased, weakness and agility increased), owners had to be satisfied with the treatment outcome and post-ACTH cortisol concentrations had to be <138 nmol/L. Median time for urinary sample collection during trilostane therapy was 11 months (range: 6-40 months) after diagnosis.

#### Statistical analysis

Data were analyzed with non-parametric statistical methods (GraphPad Prism6, Graph Pad Software, San Diego, CA, USA, SPSS 22.0 for Windows, SPSS Inc., Chicago, IL USA). Ranges and median values are reported. The Wilcoxon signed rank test was used for comparisons between different time points, and the Mann-Whitney U-Test for comparisons between different groups. Linear correlations were calculated by Spearman’s non-parametric correlation. Values of *p* < 0.05 were considered significant.

Preliminary reference intervals for the urinary catecholamines and metanephrines were generated by the nonparametric method of percentile estimates with confidence intervals to determine the central 95th percentile interval (i.e. 2.5 through 97.5th percentile range) for results from clinically normal dogs [17]. Reference intervals thus determined were: epinephrine: creatinine = 1-18; norepinephrine: creatinine = 1-19; metanephrine: creatinine = 12-255; normetanephrine: creatinine = 14-123.

## Results

### Catecholamines and metanephrines before and during therapy

**Fig. 1:**
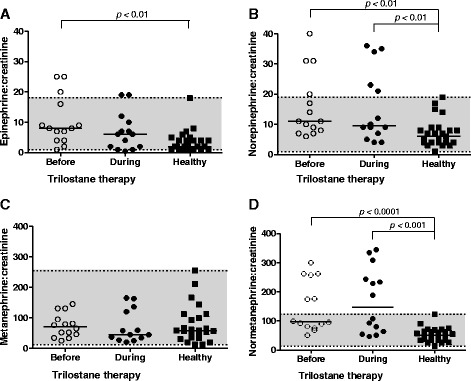
Urinary epinephrine (**a**), norepinephrine (**b**), metanephrine (**c**) and normetanephrine:creatinine (**d**) in dogs with hypercortisolism *before* (open circles) and *during* (closed circles) trilostane therapy and in healthy dogs (squares). The horizontal bars represent the median of each group. The shaded area indicates the preliminary reference interval

Concentrations of catecholamines and metanephrines for dogs with HC before and during trilostane therapy and for healthy dogs are depicted in Fig. 1 and Table 1. In one dog the metanephrine:creatinine ratio could not be measured during trilostane therapy due to a chemical artefact.

**Table 1:** Ranges (median/SEM) of urinary catecholamines and metanephrines to creatinine ratios in dog with hypercortisolism (HC) before and during trilostane therapy and in healthy dogs

Parameters	Dogs with HC		Healthy dogs
	before therapy	during therapy	
Epinephrine:creatinine	1-25^a^ (8 / 2.1)	0.5-19 (6 / 1.7)	1-18 (2 / 0.7)
Norepinephrine:creatinine	6-40^a^ (11 / 2.9)	4-36^a^ (9.5 / 3.2)	1-19 (6 / 0.9)
Metanephrine:creatinine	25-145 (70.5 / 10.4)	20-165 (45 / 14.9)	12-255 (58 / 12.3)
Normetanephrine:creatinine	51-300^a^ (97 / 23.4)	47-345^a^ (148 / 29.9)	14-123 (51 / 4.8)

^a^significant difference compared to healthy dogs

The epinephrine, norepinephrine, and normetanephrine:creatinine ratios *before* trilostane therapy and the norepinephrine and normetanephrine:creatinine ratios *during* trilostane therapy of dogs with HC were significantly higher than those of healthy dogs (before: *p* < 0.01, < 0.01, < 0.0001, respectively; during: *p* < 0.01, < 0.001, respectively) (Fig. 1).

There was no significant decrease in the epinephrine, norepinephrine, metanephrine and normetanephrine:creatinine ratios of dogs with HC during trilostane therapy (*p* = 0.1, 0.8, 0.2, 1, respectively) (Fig. 1).

**Fig. 2:**
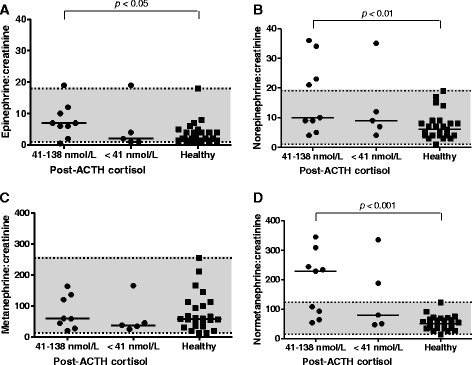
Urinary epinephrine (**a**), norepinephrine (**b**), metanephrine (**c**) and normetanephrine:creatinine (**d**) in dogs with hypercortisolism *during* trilostane therapy according to their post-ACTH cortisol concentration (41-138 nmol/L or <41 nmol/L). The horizontal bars represent the median of each group. The shaded area indicates the preliminary reference interval

Grouping dogs according to their post-ACTH cortisol concentration during trilostane therapy, revealed 9 dogs with post-ACTH cortisol between 41 and 138 nmol/L (group 1) and 5 with post-ACTH cortisol <41 nmol/L (group 2, no clinical signs of hypocortisolism evident). Concentrations of catecholamines and metanephrines for both groups during trilostane therapy and for healthy dogs are depicted in Fig. 2 and Table 2.

**Table 2:** Ranges (median/SEM) of urinary catecholamines and metanephrines to creatinine ratios in dog during trilostane therapy with post-ACTH cortisol between 41 and 138 nmol/L (group 1) and <41 nmol/L (group 2) and in healthy dogs

Parameters	Dogs with HC		Healthy dogs
	Group 1	Group 2	
Epinephrine:creatinine	0.5-19^a^ (7 / 1.8)	1-19 (2 / 3.4)	1-18 (2 / 0.7)
Norepinephrine:creatinine	4-36^a^ (10 / 4.1)	4-35 (9 / 5.6)	1-19 (6 / 0.9)
Metanephrine:creatinine	20-163 (59 / 19)	25-165 (37 / 26.2)	12-255 (58 / 12.3)
Normetanephrine:creatinine	54-345^a^ (229 / 36.3)	47-335 (80 / 55)	14-123 (51 / 4.8)

^a^significant difference compared to healthy dogs

There was no significant difference in the epinephrine, norepinephrine, metanephrine and normetanephrine:creatinine ratios between the two groups during therapy (*p* = 0.3, 0.6, 0.6, 0.3, respectively) (Fig. 2). Likewise epinephrine, norepinephrine, metanephrine and normetanephrine:creatinine ratios did not decrease significantly during treatment in both groups (group 1: *p* = 0.3, 0.7, 0.5, 0.6, respectively; group 2: *p* = 0.6, 0.5, 0.6, 0.6, respectively). Compared to healthy dogs, however, dogs with post-ACTH cortisol between 41 and 138 nmol/L still had significantly increased epinephrine, norepinephrine and normetanephrine:creatinine ratios (*p* < 0.05, < 0.01, < 0.001, respectively), whereas in dogs with post-ACTH cortisol <41 nmol/L these differences were diminished (*p* = 0.9, 0.2, 0.07, respectively) (Fig. 2).

There was no significant correlation of epinephrine, norepinephrine, metanephrine or normetanephrine:creatinine ratios with baseline or post-ACTH-stimulated cortisol or with endogenous ACTH concentrations (*n* = 6) during trilostane therapy (Table 3).

**Table 3:** Correlations between urinary catecholamines and metanephrines with cortisol and endogenous ACTH concentrations *during* trilostane therapy

Parameter	Spearman correlation coefficient / *p*-value		
	Baseline cortisol	Post-ACTH cortisol	Endogenous ACTH
Epinephrine:creatinine	0.01 / 1	0.5 / 0.05	0.2 / 0.7
Norepinephrine:creatinine	−0.18 / 0.5	0.4 / 0.2	−0.1 / 0.8
Metanephrine:creatinine	0.06 / 0.9	0.5 / 0.1	−0.2 / 0.7
Normetanephrine:creatinine	0.16 / 0.6	0.4 / 0.2	−0.8 / 0.06

## Discussion

As expected, some dogs with HC had increased urinary catecholamines and metanephrines. This is in agreement with results from previous studies, which showed that in up to 50% of dogs with HC urinary epinephrine, norepinephrine and normetanephrine:creatinine ratios are above those of control dogs [9, 10]. These findings most likely reflect the increased catecholamines synthesis (epinephrine and norepinephrine) and metabolism (conversion into metanephrine and normetanephrine) induced by glucocorticoids [3–7].

Unexpectedly, urinary catecholamines and metanephrines did not decrease significantly during trilostane therapy in dogs with HC, and thus contradicted our hypothesis. There are several possible explanations for this finding. After once-daily trilostane administration cortisol concentrations are unlikely to be reduced over a 24-h period. Trilostane is an orally administered competitive inhibitor of the 3β-hydroxysteroid dehydrogenase, which mediates the conversion of pregnenolone to progesterone in the adrenal glands [18]. Cortisol, aldosterone and androstenedione are produced from progesterone via different biochemical pathways. Trilostane inhibits the production of progesterone and therefore the synthesis of its end products [19]. It is known that the maximum inhibitory effect of trilostane on glucocorticoid production in dogs is reached 2-4 h after its administration, and that cortisol concentrations re-increase thereafter [20]. In the present study trilostane was dosed once-daily. Twice-daily therapy would lead to a more pronounced decrease in catecholamines and metanephrines concentrations than once-daily therapy, but this hypothesis has to be determined in a future study. Supporting evidence for this hypothesis is that dogs with low post-ACTH cortisol concentration (< 41 nmol/L) had a diminished increase in epinephrine, norepinephrine and normetanephrine:creatinine ratios compared to healthy dogs. In contrast, the increase in epinephrine, norepinephrine and normetanephrine:creatinine ratios persisted in the dogs with post-ACTH cortisol between 41 and 138 nmol/L.

Cortisol secretion during trilostane treatment has been evaluated in a study by Galac et al., where the authors compared urinary corticoid to creatinine ratios (UCCRs) in dogs with PDH before and during trilostane treatment after several months of optimal dosing of trilostane [21]. In that study, although UCCRs decreased significantly during therapy, they did not decrease below the upper limit of the reference range in the majority of the dogs, which might suggest that the influence of cortisol on the body system in trilostane-treated dogs persists, even in controlled dogs. Another interesting result in their study was that during long-term follow-up, a decrease in the UCCR below the upper limit of the reference range was associated with hypocortisolism. This seems similar to our results, where, as mentioned above, only dogs with a post-ACTH cortisol <41 nmol/L had urinary catecholamines and metenaphrines comparable to those of healthy dogs.

Another explanation for the failure to demonstrate a reduction in urinary catecholamines and metanephrines during trilostane therapy might be related to the blood supply of the adrenal medulla. The adrenal medulla gets its main blood supply through the capillaries of the cortex via the cortical-medullary portal system [1]. Due to this vascular system, the adrenal medulla gets in contact with glucocorticoids. In dogs with HC, even though controlled during trilostane therapy, the glucocorticoid concentration, which reaches the adrenal medulla through the portal system, is probably still much higher than under physiologic conditions.

Plasma free catecholamines and metanephrines or urinary catecholamines and metanephrines:creatinine ratios are used to diagnose canine pheochromocytomas [22]. Clinical signs usually result from secretion of excessive amounts of catecholamines, and only rarely from the space-occupying or invasive nature of the tumor [23–29]. Hypercortisolism is one of the most important differential diagnoses of pheochromocytoma. Both diseases may have similar clinical signs (weakness, polyuria, polydipsia, panting) as well as similar ultrasonographic features; in rare cases they can even occur concurrently [10]. In dogs, measurement of plasma free normetanephrine and urinary normetanephrine:creatinine ratio have been shown to be the most reliable biochemical test for diagnosing pheochromocytoma, even in cases in which the two diseases occur concurrently [9, 10, 13, 30]. Metanephrine to creatinine ratios were also significantly higher in dogs with pheochromocytoma compared to those of dogs with HC, but a considerable overlap existed [10]. This is similar to results described in human medicine, where normetanephrine concentrations have the highest sensitivity and specificity in diagnosing pheochromocytoma [31, 32]. Most likely, this reflects the fact that the vast majority of human and canine pheochromocytomas predominantly produce norepinephrine, which is metabolized to normetanephrine [10, 30, 31, 33]. Our study shows that dogs with HC treated with once-daily trilostane still have increased urinary concentrations of catecholamines and metanephrines and that diagnosing pheochromocytoma in these dogs might be challenging.

A limitation of the study is its rather low sample size. Another potential limitation is the lack of histopathology to exclude concurrent pheochromocytoma. However, only post mortem histopathology would reliably exclude concurrent pheochromocytoma, which is difficult to obtain in a clinical setting with medically treated patients. To minimize the risk of concurrent pheochromocytoma, we included only dogs with no obvious adrenal mass and with good clinical control after 6 months of trilostane therapy. Even though all dogs included seemed clinically well controlled, some dogs might have been overdosed according to their post-ACTH cortisol value (< 41 nmol/L). This lack of a proven gold standard test is another limitation of studies evaluating dogs during trilostane therapy.

## Conclusions

Although dogs with HC can be controlled during once-daily trilostane therapy, it is likely that urinary concentrations of catecholamines and metanephrines remain increased, reflecting a constant influence of the endogenously increased cortisol concentration in these dogs.

## Abbreviations

HC: hypercortisolism
HCl: hydrochloric acid
LDDS test: low-dose dexamethasone suppression test
L-DOPA: L-dihydroxyphenylalanine
PDH: pituitary-dependent hyperadrenocorticism
PNMT: phenylethanolamin-N-Methyltransferase
UCCRs: urinary corticoid to creatinine ratio
